# Implementation of Cognitive Therapy for PTSD in routine clinical care: Effectiveness and moderators of outcome in a consecutive sample^☆^

**DOI:** 10.1016/j.brat.2013.08.006

**Published:** 2013-11

**Authors:** Anke Ehlers, Nick Grey, Jennifer Wild, Richard Stott, Sheena Liness, Alicia Deale, Rachel Handley, Idit Albert, Deborah Cullen, Ann Hackmann, John Manley, Freda McManus, Francesca Brady, Paul Salkovskis, David M. Clark

**Affiliations:** a Department of Experimental Psychology, University of Oxford, South Parks Road, Oxford OX1 3UD, UK; b Oxford Cognitive Health Clinical Research Facility, UK; c National Institute for Health Research (NIHR) Mental Health Biomedical Research Centre at South London and Maudsley NHS Foundation Trust and King's College London, UK; d Department of Psychiatry, University of Oxford, UK

**Keywords:** Posttraumatic stress disorder, Cognitive behavior therapy, Cognitive therapy, Predictors of outcome, Dissemination, Implementation, Treatment effectiveness

## Abstract

**Objective:**

Trauma-focused psychological treatments are recommended as first-line treatments for Posttraumatic Stress Disorder (PTSD), but clinicians may be concerned that the good outcomes observed in randomized controlled trials (RCTs) may not generalize to the wide range of traumas and presentations seen in clinical practice. This study investigated whether *Cognitive Therapy for PTSD* (CT-PTSD) can be effectively implemented into a UK National Health Service Outpatient Clinic serving a defined ethnically mixed urban catchment area.

**Method:**

A consecutive sample of 330 patients with PTSD (age 17–83) following a wide range of traumas were treated by 34 therapists, who received training and supervision in CT-PTSD. Pre and post treatment data (PTSD symptoms, anxiety, depression) were collected for all patients, including dropouts. Hierarchical linear modeling investigated candidate moderators of outcome and therapist effects.

**Results:**

CT-PTSD was well tolerated and led to very large improvement in PTSD symptoms, depression and anxiety. The majority of patients showed reliable improvement/clinically significant change: intent-to-treat: 78.8%/57.3%; completer: 84.5%/65.1%. Dropouts and unreliable attenders had worse outcome. Statistically reliable symptom exacerbation with treatment was observed in only 1.2% of patients. Treatment gains were maintained during follow-up (*M* = 280 days, *n* = 220). Few of the selection criteria used in some RCTs, demographic, diagnostic and trauma characteristics moderated treatment outcome, and only social problems and needing treatment for multiple traumas showed unique moderation effects. There were no random effects of therapist on symptom improvement, but therapists who were inexperienced in CT-PTSD had more dropouts than those with greater experience.

**Conclusions:**

The results support the effectiveness of CT-PTSD and suggest that trauma-focused cognitive behavior therapy can be successfully implemented in routine clinical services treating patients with a wide range of traumas.

## Highlights

• Cognitive Therapy for PTSD can be effectively implemented in routine clinical care.
• The intent-to-treat analysis showed very large treatment effects.
• Patients found the treatment acceptable, and the majority showed clinically significant change.
• Few patient characteristics moderated treatment outcome.
• Inexperienced therapists had more dropouts.

A substantial number of randomized controlled trials (RCTs) have established the efficacy of trauma-focused cognitive behavioral treatments (TF-CBT) in posttraumatic stress disorder (PTSD) (for reviews see Australian Centre for Posttraumatic Mental Health, 2007; Bisson et al., 2007; Bradley, Greene, Russ, Dutra, & Westen, 2005; Kitchner, Roberts, Wilcox, & Bisson, 2012; Powers, Halpern, Ferenschak, Gillihan, & Foa, 2010; Stein et al., 2009). These RCTs have shown very large effect sizes in treating PTSD symptoms and associated symptoms of depression and anxiety for a range of TF-CBT programs. There is as yet less evidence on how effective such treatment programs are when applied in routine clinical settings. Clinicians are often concerned that that the good outcomes observed in RCTs may not generalize to the wide range of traumas and presentations seen in clinical practice.

## Do the effects of TF-CBT programs generalize to routine clinical care?

Several factors are conceivable that could potentially limit the extent to which the treatment effects observed in RCTs generalize to patients seen in routine clinical practice. Although most RCTs studied clinically pertinent samples with moderate to severe PTSD and associated comorbid conditions, they applied certain inclusion and exclusion criteria. The selection may influence outcome, for example, by increasing the average size of improvement by requiring a minimum severity or by excluding difficult-to-treat patients. One of these potential factors is that many RCTs selected patients who suffered from discrete traumas such as physical or sexual assault or traffic accidents (but may have also experienced additional other traumas, e.g., Bryant, Moulds, Guthrie, Dang, & Nixon, 2003; Ehlers et al., 2003; Foa et al., 2005; Resick, Nishith, Weaver, Astin, & Feuer, 2002; Schnurr et al, 2007), whereas in clinical practice patients may require treatment for wider range of traumas including prolonged and multiple traumatic events. It remains unclear whether the exclusion of certain demographic groups such as men, people older than 65 years of age, or comorbid conditions such as borderline personality disorder influences the overall treatment effects. Second, there have been concerns about a possible risk of symptom exacerbation with exposure to trauma memories (e.g., Tarrier et al., 1999). Although initial reports have found symptom exacerbation to be uncommon in RCT samples (e.g., Foa, Zoellner, Feeny, Hembree, & Alvarez-Conrad, 2002; Hackmann, Ehlers, Speckens, & Clark, 2004), clinicians may be concerned that this problem may be more common in patients seen in routine clinical care. A third concern relates to treatment dropouts. Many of the earlier RCTs reported completer-only analyses. If dropout rates are substantial, completer analyses may overestimate the efficacy of treatments. Some RCTs have observed high dropout rates of between 25 and 43% with trauma-focused PTSD treatments in RCTs (e.g., Foa et al., 2005; Power et al., 2002; Resick et al., 2002; Schnurr et al, 2007), although the average dropout rate may not be higher than for other PTSD treatments (Hembree et al., 2003). Fourth, in RCTs treatment is usually delivered by therapists who receive specialized training and supervision in TF-CBT, and clinicians with less training and supervision may find it difficult to replicate their results. Thus, there is a need to empirically investigate how well the excellent outcomes of TF-CBT observed in RCTs can be replicated in routine clinical settings where patients are not selected for RCT suitability and treatment is delivered by therapists with a range of prior experience with TF-CBT.

Preliminary evidence suggests that TF-CBT programs can be successfully implemented in routine clinical services (for reviews see Cohen & Mannarino, 2008; Stewart & Chambless, 2009). Foa et al.'s (2005) RCT of *Prolonged Exposure* for sexual assault survivors found equivalent outcomes for expert therapists and newly trained therapists working in a community center. Karlin et al. (2010) reported that veterans treated with *Prolonged Exposure* or *Cognitive Processing Therapy* following an extensive therapist training program implemented in the Veteran Health Administration showed a 30% decrease in PTSD symptoms in completer analyses (see also Monson et al., 2006; Tuerk et al., 2011). Levitt, Malta, Martin, Davis, and Cloitre (2007) and Brewin et al. (2010) reported large improvements in outreach programs for survivors of 9/11 and the London bombings who suffered from PTSD.

Gillespie, Duffy, Hackmann, and Clark (2002) trained therapists from a range of professional backgrounds in *Cognitive Therapy for PTSD*, a version of TF-CBT that builds on Ehlers and Clark's (2000) model of PTSD. The therapists treated an unselected group of patients seeking treatment for PTSD after the Omagh bombing in Northern Ireland and achieved similarly good outcomes as those observed in RCTs. Duffy, Gillespie, and Clark (2007) further successfully disseminated this treatment to an unselected group of patients who had experienced traumas in connection with the civil conflict in Northern Ireland, the majority of whom had experienced multiple traumatic events.

Whilst these initial studies evaluating the effectiveness of TF-CBT for PTSD are promising, they are limited in number, and further studies of larger samples of unselected patients with PTSD following the wide range of traumatic events seen in clinical settings are needed to determine the effectiveness of TF-CBT programs. The present study describes treatment outcomes of consecutive referrals to a National Health Service outpatient clinic treated with CT-PTSD. The clinic was newly opened in April 2001 and thus provided an opportunity to train new therapists in delivering this treatment, and to study treatment effectiveness, moderators of treatment outcome and possible therapist effects in a consecutive patient sample from a defined catchment area.

## Moderators of treatment effectiveness

The study investigated candidate moderators of the effectiveness of TF-CBT in routine clinical settings. We were interested in whether selection criteria for randomized controlled trials actually predict treatment response, and whether other aspects of clinical history, comorbidity or trauma history moderate treatment outcome. Kraemer, Wilson, Fairburn, and Agras (2002) distinguish two types of predictors of outcome. *Nonspecific predictors of outcome* influence the overall severity of symptoms, but do not influence the slope of treatment-induced improvement. Some TF-CBT studies have correlated candidate predictors with symptom severity at the end of therapy and have generally found that patients with more severe symptoms of PTSD and depression at the beginning of treatment have more remaining symptoms at the end of treatment (e.g., Blanchard et al., 2003; van Emmerik, Kamphuis, Noordhof, & Emmelkamp, 2011; van Minnen, Arntz, & Keijsers, 2002; Schulz, Resick, Huber, & Griffin, 2006). A *moderator* of treatment effectiveness is a variable that influences the slope of improvement (Kraemer et al., 2002). Several studies of TF-CBT attempted to identify moderators of treatment response in RCTs (e.g., Ehlers, Clark, Hackmann, McManus, & Fennell, 2005; Feeny, Zoellner, & Foa, 2002; Kubany et al., 2004; Rizvi, Vogt, & Resick, 2009; Tarrier, Sommerfield, Pilgrim, & Faragher, 2000; van Emmerik et al., 2011) and routine clinic samples (e.g., Gillespie et al., 2002; Richardson, Elhai, & Sareen, 2011; Rosenkranz & Muller, 2011; van Minnen et al., 2002). The results were often inconsistent and few moderators have been identified. Variables that were shown in some studies to be associated with less favorable treatment response included

• *demographic variables* such as male sex (Blain, Galovski, & Robinson, 2010), younger age (Rizvi et al., 2009; Taylor, Fedoroff, & Koch, 1999), higher level of education (Ehlers et al., 2005) or ethnic minority (Walling, Suvak, Howard, Taft, & Murphy, 2012);
• *comorbidity* with other *anxiety disorders* or high symptoms of anxiety and arousal (Rosenkranz & Muller, 2011; Tarrier et al., 2000; but see van Minnen et al., 2002; Richardson et al., 2011; for negative findings); with *depression or suicidal ideation* (Duffy et al., 2007; Tarrier et al., 2000; but see van Minnen et al., 2002; Richardson et al., 2011; for negative findings); *substance abuse* (van Minnen et al., 2002; but see Richardson et al., 2011; for negative findings); personality disorders (Clarke, Rizvi, & Resick, 2008; Feeny et al., 2002); use of *psychotropic medication* (van Minnen et al., 2002), and *permanent physical disability* resulting from the trauma (Gillespie et al., 2002; but see Duffy et al., 2007; for negative findings);
• *trauma characteristics* such as multiple trauma (van Minnen et al., 2002), childhood trauma (van Minnen et al., 2002; but also see Jaycox, Foa, & Morral, 1998; for negative findings), interpersonal trauma committed by a perpetrator (van Minnen et al., 2002), longer time since the trauma (Duffy et al., 2007; but see Ehlers et al., 2005; Rizvi et al., 2009; for negative findings).

## Therapist effects

In a meta-analysis of psychotherapy outcome studies, Crits-Christoph et al. (1991) found that on average 8.6% of the variance in outcome were due to random therapist effects. Greater therapist effects were found when no treatment manual was used and therapists were inexperienced. More recent studies are consistent with this pattern of results. Wampold and Brown (2005) estimated that about 5% of the variation in outcome of 6146 patients with different diagnoses treated in managed care was due to therapists. Similarly, Lutz, Leon, Martinovich, Lyons, and Stiles (2007) investigated outcomes of patients in managed care treated by therapists of different professional backgrounds and orientations and estimated that 8% of the variance in outcome could be attributed to therapists. Other recent studies investigated more homogeneous samples of clients with a particular disorder who were treated according to a particular protocol and found no significant effects of therapist on outcome (e.g., Cella, Stahl, Reme, & Chalder, 2011; Wilson, Wilfley, Agras, & Bryson, 2011). In PTSD, there is as yet little data on therapist effects. In RCTs, Ehlers et al. (2003, 2005, see Baldwin et al., 2011) and Kubany et al. (2004) found no therapist effects, while Duffy et al. (2007) reported significantly worse outcome for one therapist who was inexperienced in delivering the treatment protocol.

## Aims of the study

This study had the following aims:

(1) to assess the effectiveness of CT-PTSD in unselected patients referred to a National Health Service outpatient clinic,
(2) to assess treatment response of patients who do not complete treatment,
(3) to investigate whether candidate diagnostic variables, demographic variables and aspects of trauma history moderate treatment response, and
(4) to explore therapist effects on treatment outcome.

## Method

### Clinical setting and patients

The Centre for Anxiety Disorders and Trauma, Maudsley Hospital, UK, is an outpatient clinic specializing in the treatment of anxiety disorders in adults. It was opened in April 2001 and is part of the British National Health Service, receiving referrals from General Practitioners and Community Mental Health Teams. The clinic offers assessment and treatment for survivors of trauma in adulthood who suffer from PTSD. It serves a population of about 867,000 people living in the South London Boroughs of Southwark, Lewisham and Lambeth. These areas have substantially higher rates of social deprivation, crime, and a greater proportion of ethnic minorities than the UK average.

**Fig. 1:**
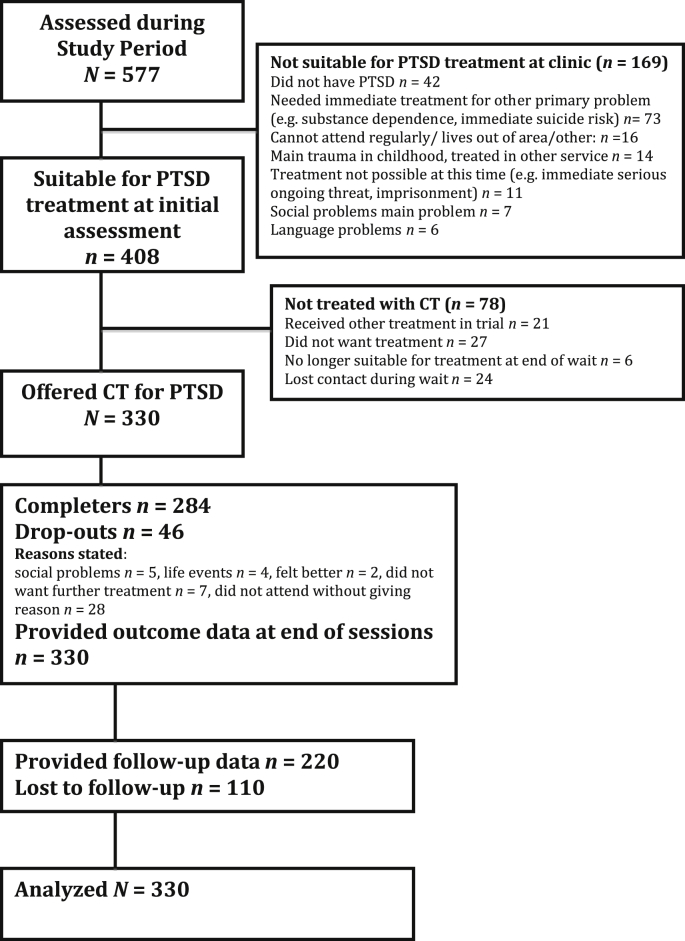
Patient flow.

The present study included all consecutive patients who were referred for assessment for possible PTSD between April 2001 and August 2008. The study was approved by the local research ethics committee. Fig. 1 shows that 577 patients completed the assessment, and 408 were suitable for trauma-focused treatment. The main reasons for not being suitable at the time of assessment were that the patient did not have PTSD (*n* = 42), or first needed treatment for another primary problem such as alcohol dependence or immediate suicide risk (*n* = 73). The main reasons for not being offered CT-PTSD despite suitability for treatment (*n* = 78) were that the patient did not want treatment (*n* = 27) or participated in a trial where they received another psychological treatment (*n* = 21). A total of 330 patients were offered a course of CT-PTSD. Data for initial assessment and the last treatment session were available for all patients, including dropouts. Two thirds (*n* = 220) of the patients provided follow-up data.

Fifty-six percent (*n* = 185) of the 330 patients in the intent-to-treat sample were female. Their ages ranged from 18 to 83 years, *M* = 38.8, *SD* = 11.5. A large proportion (43.7%) of the patients were from ethnic minorities, and either unemployed (33.7%), or on disability/retired (8.7%) or sick leave (16.0%) because of their symptoms. The majority (42.8%) was single, 37.7% were married/cohabitating with a partner, and 19.5% were divorced/widowed. The majority (43.8%) had completed mandatory school education up to age 16 (GCSE or equivalent), 18% had not completed school exams, 15% had taken higher school exams at age 18 (A level), and 23.2% had attended university.

The most common type of trauma addressed in treatment was interpersonal violence (including physical assault, sexual assault, terrorist attack and torture, 56.7%); followed by accidents or disaster (22.4%), traumatic death/harm to others (9.5%), and other traumas (11.2%). In 22.5% of patients, the trauma had involved the death of another person. The main traumas that were addressed in treatment had happened between 3 and 360 months ago, *M* = 36.5, *SD* = 56.9. Most patients (82.0%) had been injured during their traumas, and 21.5% had suffered permanent physical disabilities or loss of function due to the trauma. A third of the patients (33.7%) had received previous treatment for their PTSD, and 9.1% had had a previous course of trauma-focused CBT. The majority (63.0%) reported a history of further traumas, and 17.8% reported a history of childhood abuse.

Comorbidity with other disorders was common; 75.8% met diagnostic criteria for at least one current comorbid Axis 1 disorders, and 29.1% met criteria for Axis II disorders. The most common comorbid Axis 1 conditions were mood disorders (50.9%), other anxiety disorders (42.7%) and substance abuse (22.1%). Nearly half (48.5%) reported current suicidal ideation, and 14.2% reported past suicide attempts. A history of major depression was common (69.7%), and a significant minority (18.8%) reported a history of substance dependence. The mean number of comorbid disorders was 2.2, *SD* = 2.1.

Patients taking psychotropic medication (*n* = 132, 40.0%) were asked to remain on a stable dose for two months before treatment started and to stay on the same dose for the duration of treatment, and the majority of patients reported that they followed this advice. The majority (54.5%) of these patients were taking SSRIs, 16.3% tricyclic antidepressants, 4.9% benzodiazepines, 6.5% hypnotics. and 17.9% other medication.

Patients who provided follow-up data (*n* = 220) were comparable to those who did not (*n* = 110) on most demographic, diagnostic and trauma history characteristics, but had higher education levels, *χ*^2^ (3, *n* = 306) = 11.91, *p* = .008; were more likely to be employed, *χ*^2^ (1, *n* = 312) = 4.91, *p* = .027, and less likely to have comorbid mood disorders, *χ*^2^ (1, *n* = 330) = 14.77, *p* < .001, current substance abuse, *χ*^2^ (1, *n* = 330) = 5.62, *p* < .018, or personality disorders, *χ*^2^ (1, *n* = 330) = 4.99, *p* = .025.

### Therapists

Therapists included both qualified clinicians and therapists in training. A total of 34 different therapists with a wide range of prior experience in CBT treated patients during the study period. This included clinical psychologists (qualified *n* = 15, 44.1%; trainees = 11, 32.3%), nurse therapists (*n* = 3, 8.8%) and psychiatrists (trainees, *n* = 5, 14.7%). Each therapist participated in a two-day workshop in CT-PTSD. They then received close individual supervision in treating their first few cases and had the opportunity to act as co-therapists with a trained therapist for at least one case. Thereafter, cases were discussed in weekly CT-PTSD focused group supervision. Data for all patients including training cases were included in the data analysis. Two aspects of therapist experience were coded: first, whether the therapist was a trainee or a staff therapist, and second, the therapists' experience with CT-PTSD (experienced was defined as having treated more than 12 patients with CT-PTSD).

### Treatment

CT-PTSD is based on Ehlers and Clark's (2000) model of PTSD and targets three factors specified in this model. It is suggested that people with PTSD perceive a serious current threat which has two sources, excessively negative appraisals of the trauma and/or its sequelae and characteristics of trauma memories that lead to reexperiencing symptoms. The problem is maintained by cognitive strategies and behaviors (such as thought suppression, rumination, and safety-seeking behaviors) that are intended to reduce the sense of current threat, but maintain the problem by preventing change in the appraisals or trauma memory, and/or by increasing symptoms. Details of the treatment procedures are found in Clark and Ehlers (2004), Ehlers et al. (2005) and Ehlers et al. (2010, http://oxcadat.psy.ox.ac.uk/downloads/CT-PTSD%20Treatment%20Procedures.pdf/view). Treatment was conducted in English in individual treatment sessions. Patients received a mean of *M* = 10.6 weekly treatment sessions, *SD* = 5.0, and *M* = 2.0 monthly booster sessions, *SD* = 3.0, similar to previous trials (Ehlers et al., 2003, 2005). Therapists kept detailed notes about each treatment session, and an independent rater rated the extent to which the session focused on the PTSD treatment model, on a scale from 1 to 3 (1 = *mainly followed trauma-focused protocol*, 2 = *equal focus on trauma-focused protocol and other problems*, 3 = *main focus on other problems*). The mean rating for all sessions was *M* = 1.35, *SD* = 0.39, and for 90.1% of the patients treatment mainly focused on the PTSD treatment protocol (mean rating of below 2). The most common other problems addressed in the sessions were comorbid disorders, and other stressors such as social problems (e.g., financial, housing, legal issues) or physical health problems.

## Measures

Self-reports of symptom severity were taken at initial assessment, at the first and last treatment session, and at follow-up (mean 280 days).

### Severity of PTSD symptoms

The primary outcome measure was the change in PTSD symptoms. Patients completed the Posttraumatic Diagnostic Scale (PDS, Foa, Cashman, Jaycox, & Perry, 1997). The PDS asks patients to rate how often they were bothered by each of the PTSD symptoms specified in DSM-IV ranging from 0 = *never* to 3 = *5 times per week or more/almost always*. The PDS yields a sum score measuring the overall severity of PTSD symptoms. Foa et al. (1997) showed that the self-report questionnaire has good reliability and concurrent validity with other PTSD measures. Internal consistency in this sample was *α* = .85.

### Depression and anxiety

Symptoms of anxiety and depression were secondary outcome measures. Patients completed the Beck Anxiety Inventory (BAI, Beck & Steer, 1993a) and the Beck Depression Inventory (BDI, Beck & Steer, 1993b), standard 21-item self-report measures with high reliability and validity. Internal consistencies in this sample were *α* = .92 and *α* = .90, respectively.

### Candidate moderators

At initial assessments, clinicians conducted the *Structured Clinical Interview for DSM-IV* (SCID) to assess Axis I (First, Spitzer, Gibbon, & Williams, 1996) and II diagnoses (First, Gibbon, Spitzer, & Williams, 1995). Interrater-reliability for PTSD (determined from a random selection of 37 audiotapes of the interviews) was *κ* = 0.95. Clinicians also determined in the interview what the patient's main problem was and assessed physical consequences of the trauma (disability, chronic pain) and the patients' treatment and trauma history (adapted from the Clinician Administered PTSD Scale, Blake et al., 1995). Patients provided demographic information.

Four groups of potential moderators were extracted on the basis of the literature reviewed above.

1. *Selection criteria used in some previous RCTs*: male sex; age; patient does not meet full DSM-IV criteria (American Psychiatric Association, 1994) and only meets ICD-10 (World Health Organization, 2010) PTSD criteria; PTSD is not the main problem (i.e., another disorder is so severe that it needs principal concurrent treatment in its own right, e.g., very severe depression or agoraphobia); borderline personality disorder; current substance abuse; current PTSD is linked to multiple traumas; history of childhood abuse; no memory for the trauma; patient had a previous course of trauma-focused CBT for PTSD.
2. *Demographic variables*: ethnic group; education; social problems (defined as one or more of unemployment, financial hardship, housing problems); relationship status (never married/lived with partner vs. married, cohabiting, widowed or divorced); ongoing legal proceedings, any previous treatment for PTSD.
3. *Comorbidity*: current comorbid anxiety disorder, mood disorders, other axis 1 disorders, personality disorder, suicidal ideation, taking psychotropic medication; physical disability resulting from trauma, chronic pain; history of major depression, substance dependence, or suicide attempts.
4. *Aspects of trauma history*: Trauma type (interpersonal trauma vs other); trauma involved death of other person; injured in trauma; months since main trauma; history of other traumas; total number of traumas experienced.

### Treatment variables

Three aspects of the course of treatment were coded from the session notes to test possible effects of the moderators on treatment delivery.

1. Dropout: This was defined as attending less than 8 sessions, unless the earlier termination was determined in agreement with the therapist. This criterion was chosen because UK treatment guidelines (National Institute for Health and Clinical Excellence, 2005) recommend 8 sessions as an adequate trial of trauma-focused treatments for PTSD on the basis of prior research.
2. Attendance: Therapists indicated in the session notes whether or not the patient was often late or missed appointments without notifying the therapist.
3. Trauma focus: the degree to which treatment followed the trauma-focused PTSD treatment protocol, as above.

## Data analysis

### Measures of treatment response

The main outcome variable was the change in PDS scores with treatment. Several further measures of treatment response were calculated for comparability with previous studies.

#### Reliable improvement and exacerbation

Reliable change thresholds for the PDS were calculated by Foa et al. (2002) on the basis of the retest reliability and standard deviation of the scale. Reliable improvement and exacerbation are decreases/increases in PDS scores of greater than 6.15, respectively.

#### Clinically significant treatment response

Clinical significant response was defined as in Jacobson and Truax (1991). Patients had to show a reliable improvement and their score at the end of treatment had to be lower than the halfway point between 2 SD below the patients' scores at the beginning of treatment, and 2 SD above the mean of a sample of 466 traumatized people without PTSD from the same catchment area (*M* = 7.22, SD = 7.75), i.e., lower than 19.775. This criterion is similar to the PDS cut-offs between clinical and nonclinical presentations established by Ehring, Kleim, Clark, Foa, and Ehlers (2007) on the basis of agreement with structured diagnostic interviews.

#### Effect size

Treatment effect sizes for changes in symptom scores between the pre treatment assessment and final treatment session were calculated using Cohen's *d* statistic (Cohen, 1988). As other studies vary in whether effect sizes are reported in relation to pooled pre-post standard deviations, or pre-treatment standard deviations, we report both for comparison.

### Statistical analysis

The Statistical Package for Social Sciences, version 20, was used for data analysis. Hierarchical linear modeling investigated the effect of candidate moderators and therapist effects on the degree of improvement in the PDS with treatment, following guidelines by Heck, Thomas, and Tabata (2010). This analysis uses data from all patients. All variables were centered for this analysis (Kraemer et al., 2002). At Level 1, the effects of repeated observations nested within patients were considered. Model 1 modeled the slope of improvement in PDS scores from pre treatment to post treatment to follow-up and tested random slopes and intercepts for patients. Linear and quadratic changes in PDS scores with time were fitted, using an autoregressive covariance structure at Level 1. At Level 2, patient characteristics that may influence outcome were first added individually (because of some missing data on some of the variables) to the model to test for main effects on PDS scores (indicating nonspecific prediction of symptom severity, Kraemer et al., 2002) and interactions with the slope of improvement (moderator effects, Kraemer et al., 2002). Next, variables that showed significant interactions were combined into an overall Model 2 to determine unique moderators of slope of improvement. At Level 3 (therapist effects), random slopes and intercepts for therapists were included in Model 3. In Model 4, the two measures of therapist experience were added, trainee versus staff therapist and experienced versus inexperienced in delivering CT-PTSD.

## Results

### Overall effectiveness of treatment

There was no significant change in PTSD symptoms during the wait period between assessment and treatment, *M* = 97.1 days, *SD* = 77.0, PDS scores *M* = 33.88, *SD* = 8.67 to *M* = 33.15, *SD* = 9.25, *F*(1, 275) = 2.71, *p* = .101, *d* = 0.08.

**Fig. 2:**
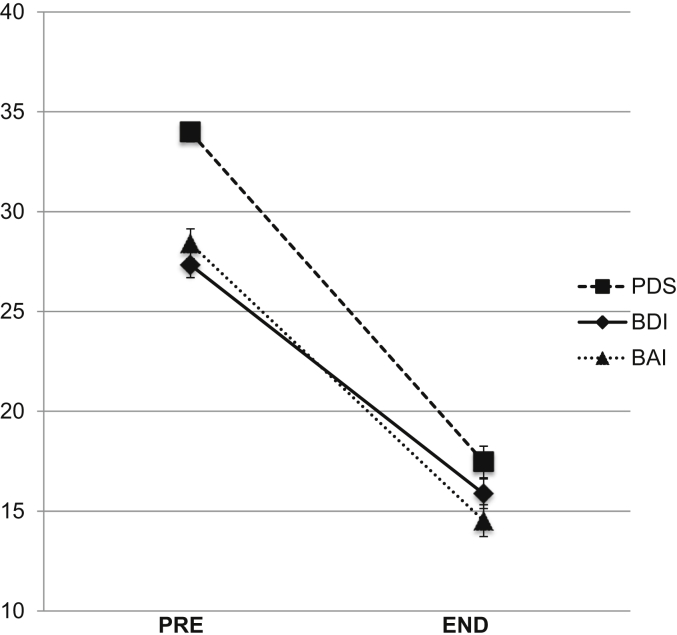
Intent-to-treat outcome for patients who were offered treatment (*n* = 330). PDS = Posttraumatic Diagnostic Scale (*n* = 330); BDI = Beck Depression Inventory (*n* = 320); BAI = Beck Anxiety Inventory (*n* = 321).

Table 2 shows the PTSD symptom scores (PDS) at initial assessment and the last treatment session for the intent-to-treat sample of 330 patients (see also Fig. 2). Patients received a mean of 12.57 sessions (SD = 6.51). Patients showed very large improvement in PTSD symptom severity with treatment. Two hundred sixty patients (78.8%) showed a reliable improvement (Foa et al., 2002). The mean percent change in PTSD symptoms was 50.4%, *SD* = 40.38. The majority of patients (*n* = 189, 57.3%) showed a clinically significant change. Clinically significant change was associated with greater trauma focus of the sessions, *M* = 1.31, *SD* = 0.37 versus *M* = 1.41, *SD* = 0.42, *F*(1, 322) = 5.51, *p* = .020, *d* = 0.17.

In treatment completers, PDS scores decreased from *M* = 33.83, *SD* = 8.67 to *M* = 15.18, *SD* = 13.65, *d* = 1.63 for pooled *SD* and *d* = 2.15 for pre-treatment *SD*, and the mean change in PDS scores was 57.95%, *SD* = 34.95. A reliable change in PDS scores was observed in 240 (84.5%) of the completers, and a clinically significant change in 185 (65.1%).

### Analysis of dropouts and attendance

The overall dropout rate was 13.9%, 46 of 330 patients. Of these, 6 patients (13%) dropped out after 1 session, 11 (23.9%) after 2 sessions, 8 (17.4%) after 3 sessions, 4 (8.7%) after 4 sessions, 7 (15.2%) after 5 sessions, 4 (8.7%) after 6 sessions, and 6 (13.0%) after 7 sessions. Patients who dropped out had waited longer for treatment *t (*327) = 4.89, *p* = .005. Among dropouts, only 8.7% (4/46) showed a clinically significant treatment response, compared to 66.9% (190/94) of treatment completers, *χ*^2^ (1, n = 330) = 55.36, *p* < .001.

Twenty-one percent of the patients (*n* = 71) were classified as unreliable attenders. This variable was independent of dropout status, *C* = 0.049. Unreliable attenders were less likely to show clinically significant change than patients who attended regularly, 42.3% versus 63.3%, *χ*^2^ (1, n = 330) = 10.21, *p* = .001.

### Symptom exacerbation

Fourteen patients (4.3%) had reliable increases on the PDS between initial assessment and the end of treatment. For 10 of the 14 patients (71.4%), the reliable increase had already occurred during the wait period between the initial assessment and the first session. Thus, only 4 (1.2%) patients showed reliable exacerbation in symptom severity during treatment.

### Improvement in other symptoms

Table 1 shows the changes in depressive (BDI) and anxiety (BAI) symptoms with treatment. Patients showed large improvement in these secondary outcomes.

### Stability of treatment effects

Two thirds of the patients (*n* = 220) provided follow-up data. The mean duration of the follow-up was 280.1 days, *SD* = 177.7. There were no significant changes in symptom scores between the end of treatment and follow-up, PDS: *F*(1, 217) = 1.01, *p* = .317, *η*^*2*^ = 0.005; BDI: *F*(1, 211) = 0.64, *p* = .425, *η*^*2*^ = 0.003; BAI, time effect *F*(1, 212) = 0.28, *p* = .595, *η*^*2*^ = 0.001.

### Moderators of treatment response

To reduce the number of potential predictors, candidate moderators were first considered individually (see Table 2). Few of the RCT selection criteria, comorbidity, trauma and demographic characteristics selected from the literature predicted outcome. Significant univariate moderation effects were found for 8 predictors: PTSD is not the main problem, patient needs treatment for multiple traumas, social problems, relationship status, comorbid mood disorder, history of suicide attempts, history of substance dependence, longer time since the main trauma were associated with somewhat less improvement. Several other variables predicted a greater overall severity of PTSD symptoms, but not the slope of improvement (non-specific predictors). These included meeting DSM-IV criteria for PTSD, aspects of comorbidity (current substance abuse, suicidal ideation, axis 1 disorder other than anxiety or mood disorder, comorbid personality disorder, chronic pain, physical disability due to the trauma, history of major depression), taking psychotropic medication, and a higher level of education.

Table 3 shows the results of the hierarchical modeling analyses for four models of increasing complexity, which are shown in separate columns. Overall model fits and random effects for patients and therapists are shown at the top of the table, and fixed effects for candidate moderators and therapist variables at the bottom. Model 1 (Level 1, random slopes and intercepts for patients and improvement in PDS scores from pre treatment to post treatment to follow-up and) showed highly significant linear and quadratic changes in PDS scores across assessment points, indicating a steep decrease in PTSD symptoms with treatment, which flattened out during follow-up. Random slopes and intercepts for patients were also highly significant.

Model 2 added patient characteristics at Level 2; analysis was restricted to the 8 candidate moderators that showed univariate moderation effects. In the multivariate analysis, unique moderation effects were found for needing treatment for multiple traumas and social problems. There was a trend for relationship status. Comorbid mood disorders and social problems were nonspecific predictors, i.e., were associated with higher scores both at the beginning and end of treatment. All other effects were nonsignificant.

### Therapist effects

In Model 3 (Table 3) random intercepts and slopes were added for therapists. There were no significant random therapist effects, and the results for patient characteristics were identical to Model 2. Model 4 added two aspects of therapist experience (staff therapist, experience with CT-PTSD) to the prediction. There was a trend for inexperienced therapists to achieve somewhat less good outcome.

### Association of moderators with treatment variables

Patients who needed treatment for multiple trauma were more likely to attend irregularly than those who were treated for one or two traumas, 33.3% versus 19.6%, *χ*^2^ (1, *n* = 330) = 4.31, *p* = .038, but did not differ in dropout rates or number of treatment sessions. Their treatment was less trauma-focused than treatment of other patients, *M* = 1.46 (*SD* = 0.37) versus *M* = 1.33 (*SD* = 0.40), *F*(1,322) = 4.28, *p* = .039, *η*^*2*^ = 0.013.

Patients who had social problems were more likely to drop out of treatment, 18.5% versus 7.2%, *χ*^2^ (1, *n* = 327) = 8.54, *p* = .003. Their treatment tended to be less trauma-focused than treatment of other patients, *M* = 1.39 (*SD* = 0.42) versus *M* = 1.30 (*SD* = 0.36), *F*(1,319) = 3.432, *p* = .065, *η*^*2*^ = 0.011. Associations with unreliable attendance (*p*. = 0.105) and lower number of sessions (*p* = .094) failed to reach significance.

Patients were more likely to drop out if the therapist was inexperienced in delivering CT-PTSD, 18.1% versus 10.3%, *χ*^2^ (1, *n* = 330) = 4.15, *p* = .042.

## Discussion

The study supports the effectiveness of *CT for PTSD* in routine clinical practice. It shows that this treatment can be successfully implemented in a National Health Service clinic serving an ethnically mixed urban catchment area, with therapists who ranged in previous experience in CBT and in treating PTSD. The clinic's catchment area was clearly defined, referral was by local family doctors and community mental health teams, and a consecutive sample was assessed, supporting the representativeness of the results. The intent-to-treat analysis showed very large effect sizes for improvement in PTSD symptoms with treatment. The effect size estimates are conservative as training cases were included in the analysis. The mean improvement in PTSD symptoms was large, 50.3% for the intent-to-treat sample, and 57.8% for completers, and the majority of patients showed clinically significant change, 57.3% and 65.1% respectively. Depression and general anxiety symptoms also showed substantial improvement with treatment.

Treatment was well tolerated. The overall dropout rate of 13.9% was low, despite the fact that the clinic served a catchment area characterized by high social deprivation and high mobility and that some of the therapists were inexperienced in delivering the treatment. It is below that observed in many RCTs (e.g., 34% Resick et al., 2002; 43% Power et al., 2002; 34% Foa et al., 2005; 38% Schnurr et al, 2007; 26% Galovski, Blain, Mott, Elwood, & Houle, 2012) and effectiveness studies of trauma-focused PTSD treatments (e.g., 36% Levitt et al., 2007; 24% and 32%; van Minnen et al., 2002). Although limited conclusions can be drawn from comparing dropout rates in different samples across countries with different health systems, it appears safe to conclude that the low dropout rates support the acceptability of CT-PTSD to patients. This study further found that patients who dropped out and those who attended irregularly had poorer outcomes, so that limiting dropout rates is likely to improve the overall effectiveness of interventions.

Consistent with previous studies (Foa et al., 2002; Hackmann et al., 2004), symptom exacerbations were only found in a small minority of patients. Interestingly, the results indicated that the exacerbation mainly occurred between assessment and the start of treatment. This result is similar to Duffy et al. (2007) who found that deterioration was more common in the wait period than during therapy. For the majority of patients, such symptom exacerbations may thus reflect the influence of other factors such as new trauma or additional stressors rather than negative effects of treatment per se.

This raises the question of what effect waiting for treatment may have on the probability of engagement with treatment. The average waiting time of about 3 months was relatively short for psychological services in the UK National Health Service at the time of the study. Overall, symptom scores were stable over the waiting period. Some patients waited longer, partly due to the availability of therapists and partly due to patient-determined factors such as scheduled surgery/physical rehabilitation, work schedules, travel or childbirth. It is unlikely that the wait for treatment contributed to the relatively low dropout rates observed in this study as patients who dropped out had waited longer than completers. This suggests that it is desirable to reduce waiting times to help reduce the risk of symptom deterioration and dropouts.

Effect sizes in the present study were comparable to those obtained by Duffy et al. (2007) with the same treatment in an unselected sample of patients with very chronic PTSD. The intent-to-treat effect sizes were somewhat smaller than those observed in some previous RCTs of CT-PTSD (Ehlers et al., 2003, 2005), but similar to or larger than those observed in intent-to-treat analyses in other RCTs of trauma-focused cognitive behavior therapy (Bryant et al., 2003; Resick et al., 2002; Schnurr et al, 2007). Given that the study reports on a consecutive intent-to-treat sample with a wide range of traumas from an ethnically diverse and socially deprived catchment area, and patients were treated by both trainees and experienced clinicians, the outcomes can be considered as encouraging.

The moderator analysis showed that many of the criteria that have sometimes been used to exclude patients from RCTs were not related to poorer outcome. The only exceptions were that patients who needed treatment for multiple traumas and those for whom PTSD was not the main clinical problem showed somewhat less improvement. Overall the results suggest that the treatment is effective in patients who do not meet all inclusion criteria for RCTs and should not be withheld from these patients (see also van Minnen et al., 2002).

Like other studies of TF-CBT programs (e.g., Richardson et al., 2011; van Minnen et al., 2002), this study found few other moderators of treatment response, further indicating that CT-PTSD is effective in a wide range of patients. Most demographic variables such as sex, age, ethnic group or education level were unrelated to treatment response. This is consistent with the results of several other studies (e.g., Richardson et al.., 2011; van Minnen et al., 2002), although some studies reported poorer outcome or larger drop-out rates for men (Blain et al., 2010). It remains to be tested whether the differences in results are due to sample differences or differences in procedures.

With the exception of mood disorders, current comorbidity did not moderate outcome, but acted as a nonspecific predictor of outcome only. High levels of depression were also associated with less favorable outcome in Duffy et al. (2007) and Tarrier et al. (2000), but not in other studies of TF-CBT (Ehlers et al., 2005; Richardson et al., 2011; van Minnen et al., 2002). One possible explanation for the discrepant results may be the range of depression severity included in the studies. The studies that reported negative findings had lower mean depression scores than those finding an effect of depression. Comorbid depression may only hamper progress in therapy if it is so severe that it affects daily activity levels and motivation to engage in the therapy assignments. The lack of a moderating effect of comorbid personality disorders including borderline personality disorder is noteworthy and consistent with other studies (Clarke et al., 2008; Feeny et al., 2002).

Longer time since the trauma, a history of suicide attempts or substance dependence, social problems, and relationship status (never having been married or lived with a partner) were associated with a somewhat less favorable treatment response. The effect of past suicide attempts replicates Tarrier et al.'s (2000) findings. These characteristics, and possibly very high levels of depression, may characterize a group of patients with high levels of demoralization and hopelessness, who may not have been fully engaged in treatment by their therapists. Time since the trauma has shown an inconsistent pattern of associations with outcome. RCTs have generally not found an effect (e.g., Ehlers et al., 2005; Resick et al., 2002), although Schnurr et al (2007) concluded that the modest treatment gains observed in their study may be related to the extreme chronicity of their sample (*M* = 23 years). Like the current study, Duffy et al. (2007) also found that longer duration since the trauma was associated with less favorable outcome. The differences in results may also be linked to the wide range of traumas included in the latter samples (for example, the inclusion of traumatic death of significant others), or the long-term effects of the trauma and/or PTSD symptoms on important life areas such as employment, significant relationships or other resources (Hobfoll, 2002). The present sample, like Duffy et al.'s (2007) included a high percentage of patients with long-term loss of resources and significant relationships. Social problems and social isolation may make it harder to overcome PTSD and may also create additional ongoing stress (see also Galovski et al., 2012). The result that patients who had never lived with a partner had somewhat worse outcome may also point to a role of poor social support and interpersonal skills in the recovery from PTSD (see also Cloitre, Koenen, Cohen, & Han, 2002).

When the significant moderators were considered together in a multivariate analysis, only social problems and needing treatment for multiple traumas emerged as unique moderators of outcome. The associations of these moderators with treatment characteristics suggest a dose–response effect. Treatment was less trauma-focused for these patients and significant time in the sessions was used to address other important problems such as comorbid disorders or social problems such as problems with state benefits or social housing, with a similar overall treatment duration of between 12 and 13 sessions. Patients with multiple trauma were less reliable in attending sessions than other patients, and those with social problems were more likely to drop out. This may have led to a less than optimal dose of treatment for each of the patients' traumas, and more treatment sessions may be necessary to further improve outcome (see also National Institute for Health and Clinical Excellence, 2005). For example, Galovski et al. (2012) found significant further treatment gains with cognitive processing therapy for patients who had not fully responded by session 12. Therapists may need to pay special attention to engaging these patients in treatment. These patients may also benefit from ancillary case management services.

When therapists were included as a random factor in the hierarchical linear model, no significant effects on treatment outcome were observed. This result is consistent with those of other studies of patients with a particular disorder who were treated with a defined treatment protocol (Baldwin et al., 2011; Cella et al., 2011; Kubany et al., 2004; Wilson et al., 2011), and with Crits-Christoph et al.'s (1991) conclusion that the use of a treatment manual reduces therapist effects. Studies of mixed patient samples treated by therapists of different theoretical orientation have tended to show larger random effects of therapists, in the range of 5–8% of the variance in outcome (Lutz et al., 2007; Wampold & Brown, 2005). These results do not necessarily contradict each other as smaller variation in treatment procedures may restrict the variance due to individual therapists. In this study, therapist experience with the specific treatment protocol showed a trend for an association with somewhat better outcome, and inexperienced therapists had more dropouts. This result is similar to Duffy et al. (2007) who found that one reason for dropouts was that some inexperienced therapists pushed patients into reliving their trauma without adequately addressing their concerns first. Training programs and supervision for novice therapists need attention on how to effectively engage patients with trauma memory work.

The study had several limitations. First, the study did not have an untreated control group and the results therefore cannot be unambiguously interpreted as therapy effects. However, several factors suggest that it is unlikely that the symptom changes represented natural recovery. Patients were referred to the clinic as they were judged by health professionals to need professional help, they had chronic, moderate to severe PTSD with a mean duration of 3 years, high comorbidity, and did not improve during the wait period before treatment started. Second, the clinic focuses on PTSD following trauma in adulthood and it remains unclear whether the results generalize to patients whose main traumas were in childhood. However, a history of childhood abuse was not predictive of poor outcome in this sample (see also Ford & Kidd, 1998). Third, although data for the intent-to-treat analysis of treatment effects were complete, the sample size was reduced at follow-up and it remains unclear whether loss to follow-up was random. Fourth, we did not have the resources to obtain fidelity or therapist competency ratings from recordings of the therapy sessions. This may have introduced error variance. However, the close supervision of all cases ensured that therapists followed the protocol. The analysis of session notes confirmed that treatment sessions mainly focused on the PTSD treatment protocol. Fifth, some of the 577 patients who were referred to the clinic for an assessment were not suitable for PTSD treatment at the time and were treated elsewhere. The most common reasons were not having PTSD (*n* = 42) and needing treatment for another problem such as substance dependence or immediate suicide risk first (*n* = 73). This pattern is to be expected as General Practitioners in the UK National Health Service have very limited time for each consultation and may ask for specialist assessment to determine the best care pathway for their patients. Nevertheless, this pattern highlights the fact that not only RCTs, but also routine clinical services have intake criteria and need to exclude some patients, and that some of the reasons for why a trauma-focused treatment is not offered may overlap. Sixth, the sample size was modest for the investigation of therapist effects and larger samples may be more sensitive in detecting therapist variables that are associated with good outcome.

In conclusion, the results support the effectiveness of CT-PTSD in a wide range of traumas and suggest that CT-PTSD can be successfully implemented in the routine care of patients with PTSD. Patients who need treatment for multiple traumas, severe comorbid disorders or social problems may benefit from extending the duration of treatment. Training new therapists in CT-PTSD or other TF-CBT protocols may benefit from special attention to engaging patients with treatment to avoid dropouts.

## Figures and Tables

**Table 1:** Intent-to-treat treatment outcome for all consecutive patients who were offered treatment including drop-outs.

Outcome measure and assessment point	All patients (*N* = 330)	
	*M*	*SD*
**PDS** pre treatment	33.98	8.77
End of treatment	17.46	14.37
***ES*** (pre – end, pooled SD)	*d* = 1.39	
***ES*** (pre – end, pre SD)	*d* = 1.88	

**BDI** pre treatment	27.33	11.75
End of treatment	15.87	13.42
***ES*** (pre – end, pooled SD)	*d* = 0.91	
***ES*** (pre – end, pre SD)	*d* = 0.98	

**BAI** pre treatment	28.39	13.62
End of treatment	14.52	14.92
***ES*** (pre – end, pooled SD)	*d* = 0.97	
***ES*** (pre – end, pre SD)	*d* = 1.02	

PDS = Posttraumatic Diagnostic Scale (*N* = 330); BDI = Beck Depression Inventory (*n* = 320); BAI = Beck Anxiety Inventory (*n* = 321); ES = Effect Size, *d* = Cohen's *d*, SD = standard deviation

**Table 2:** Overview of individual candidate predictors of outcome and fixed effects estimates from hierarchical linear modeling (in points on centered PDS scale).

Predictor	Type of effect	Size of effect (fixed effect estimate and standard error)
**Exclusion criteria used in some RCTs**		
Male sex	Not a predictor	
Age	Not a predictor	
Does not meet full DSM-IV criteria	Nonspecific predictor	−10.35 (1.68)***
PTSD not main problem	Moderator	3.31 (1.21)**
Current substance abuse	Nonspecific predictor	2.50 (1.26)*
Borderline personality disorder	Not a predictor	
Needs treatment for multiple traumas	Moderator	4.01 (1.30)**
History of childhood abuse	Not a predictor	
No memory of trauma	Not a predictor	
Previous CBT for PTSD	Not a predictor	

**Demographics**		
Ethnic minority	Not a predictor	
Lower level of education	Nonspecific predictor	−2.78 (1.03)
Social problems	Moderator and Nonspecific predictor	5.23 (1.02)*** 3.76 (0.89)***
No relationship (never married or living with partner)	Moderator	2.46 (0.93)**
Ongoing legal proceedings	Not a predictor	
Any previous treatment for PTSD	Not a predictor	

**Comorbidity**		
Current comorbid anxiety disorder	Not a predictor	
Current mood disorder	Moderator and Nonspecific predictor	1.95 (0.92)* 7.00 (0.97)***
Current other axis 1 disorder	Nonspecific predictor	4.73 (1.78)**
Any personality disorder	Nonspecific predictor	2.57 (1.15)*
Current suicidal ideation	Nonspecific predictor	5.92 (0.99)***
Taking psychotropic medication	Nonspecific predictor	5.87 (1.08)***
Chronic pain	Nonspecific predictor	3.08 (1.14)**
Physical disability due to trauma	Nonspecific predictor	2.54 (1.27)*
History of major depression	Nonspecific predictor	5.39 (1.10)***
History of substance dependence	Moderator and Nonspecific predictor	3.34 (1.19)** 3.48 (1.33)**
Past suicide attempts	Moderator and Nonspecific predictor	2.96 (1.30)* 3.34 (1.50)*
**Trauma History**		
Main trauma interpersonal	Not a predictor	
Someone died in main trauma	Not a predictor	
Injured in trauma	Not a predictor	
Months since main trauma	Moderator	1.06 (0.46)*
History of other traumas	Not a predictor	
Number of traumas	Not a predictor	
History of child abuse	Not a predictor	
Injured in trauma	Not a predictor	

*p < .05, **p < .01, ***p < .001. Nonspecific predictor: predicts symptom levels before and after treatment, but not treatment response. Moderator: predicts treatment response.

**Table 3:** Hierarchical linear modeling: Estimates of random effects (patient, therapist) and fixed effects (time, patient characteristics, therapist experience) on improvement in PTSD symptoms with therapy.

	Model 1 slope of improvement and random patient effects	Model 2 including patient characteristics	Model 3 including random therapist effects	Model 4 including therapist experience
Overall fit: AIC	6608.21	5967.25	5967.19	5951.56

**Variance-covariance estimates for random parameters**				
*Level 1: patient*				
Patient random intercept	66.46 (12.16)***	41.09 (10.44)***	37.41(11.22)***	35.39 (11.85)**
Patient random slope	40.71 (6.87)***	29.66 (5.49)***	28.11 (5.49)***	27.11 (5.46)***

*Level 2: therapist*				
Therapist random intercept			1.88 (1.84)	1.69 (1.78)
Therapist random slope			0.79 (1.29)	1.02 (1.35)

**Fixed effect estimates**				
*Level 1: time effects*				
Intercept	11.00 (0.55)***	18.90 (2.23)***	18.89 (2.26)***	18.50 (2.30)***
Linear time effect	−24.88 (1.01)***	−18.33 (2.29)***	−18.63 (2.30)***	−19.23 (2.34)***
Quadratic time effect	8.42 (0.50)***	8.38 (0.52)***	8.35 (0.52)***	8.36 (0.52)***

*Level 2: patient characteristics*				
**PTSD not main problem**				
- Nonspecific predictor		2.43 (1.37)(*)	2.28 (1.38)	2.11 (1.40)
- Moderator effect		0.71 (1.33)	0.79 (1.33)	0.44 (1.35)
**Multiple traumas need treatment**				
- Nonspecific predictor		1.99 (1.50)	1.79 (1.49)	1.69 (1.51)
- Moderator effect		3.81 (1.38)**	3.66 (1.39)**	3.25 (1.40)*
**Social problems**				
- Nonspecific predictor		3.26 (1.04)**	3.05 (1.04)**	3.17 (1.05)**
- Moderator effect		2.89 (0.94)**	2.79 (0.94)**	2.99 (0.96)**
**No relationship**				
- Nonspecific predictor		0.14 (1.00)	0.08 (1.00)	0.02(1.00)
- Moderator effect		1.68 (0.92)(*)	1.70 (0.92)(*)	1.55 (0.93)(*)
**Comorbid mood disorder**				
- Nonspecific predictor		5.85 (1.02)***	5.79 (1.01)***	5.79 (1.02)***
- Moderator effect		0.22 (0.94)	0.17 (0.94)	0.22 (0.94)
**Suicide attempts**				
- Nonspecific predictor		0.87 (1.46)	0.95 (1.47)	0.95 (1.48)
- Moderator effect		1.31 (1.35)	1.33 (1.36)	1.37 (1.36)
**History of substance dependence**				
- Nonspecific predictor		2.26 (1.32)(*)	2.39 (1.32)(*)	2.37 (1.32) (*)
- Moderator effect		1.76 (1.24)	1.77 (1.24)	1.84 (1.24)
**Months since trauma**				
- Nonspecific predictor		−0.59 (0.50)	−0.64 (0.50)	−0.60 (0.50)
- Moderator effect		0.59 (0.46)	0.59 (0.46)	0.66 (0.47)

*Level 3: therapist effects*				
**Staff therapist**				
- Nonspecific predictor				0.69 (1.49)
- Moderator effect				2.05 (1.37)
**Experienced in CT-PTSD**				
- Nonspecific predictor				−1.19 (1.24)
- Moderator effect				−2.23 (1.15) (*)

***p < .001, **p < .01, *p < .05, (*) *p* < .10.
